# The Landscape of COVID-19 Vaccination in Zimbabwe: A Narrative Review and Analysis of the Strengths, Weaknesses, Opportunities and Threats of the Programme

**DOI:** 10.3390/vaccines10020262

**Published:** 2022-02-09

**Authors:** Grant Murewanhema, Godfrey Musuka, Knowledge Denhere, Innocent Chingombe, Munyaradzi Paul Mapingure, Tafadzwa Dzinamarira

**Affiliations:** 1 Unit of Obstetrics and Gynaecology, Department of Primary Health Care Sciences, Faculty of Medicine and Health Sciences, University of Zimbabwe, Harare P.O. Box MP167, Zimbabwe; gmurewanhema@gmail.com; 2 ICAP, Columbia University, Harare P.O. Box MP167, Zimbabwe; gm2660@cumc.columbia.edu (G.M.); ic2421@cumc.columbia.edu (I.C.); mpm2189@cumc.columbia.edu (M.P.M.); 3 University of Western Cape, Cape Town 7535, South Africa; 3911203@myuwc.ac.za; 4 School of Health Systems & Public Health, University of Pretoria, Pretoria 0002, South Africa

**Keywords:** COVID-19, COVID-19 vaccination, public health policy

## Abstract

The ongoing COVID-19 pandemic brought unprecedented challenges for the population. The advent of national COVID-19 vaccination programmes was therefore welcome as a key control strategy for the COVID-19 pandemic, as evidence has shown that vaccination is the best strategy to reduce the adverse individual and population level adverse outcomes associated with infectious diseases such as COVID-19. Zimbabwe rolled out its vaccination programme in February 2021 with an ambitious target to vaccinate at least 60% of its eligible population by December 2021. However, by that time, the country was still to reach that target. To move the vaccination programme towards achieving this target, it is crucial to understand the strengths, weaknesses, opportunities and threats to the programme. We, therefore, with this narrative review, discuss some of the strengths, weaknesses, opportunities and threats to the programme since its rollout in February 2021. Though the programme has several strengths and opportunities to leverage on, we argue that among other challenges, the emergence of new variants of concern poses one of the biggest threats to local, regional and international vaccination programmes and requires concerted multistakeholder efforts to deal with. Additionally, addressing vaccine hesitancy remains as important as availing the vaccines to the population, to obtain the most benefits out of the programme.

## 1. Introduction

The World Health Organisation (WHO) instigated emergency use authorisations (EUA) for the severe acute respiratory syndrome coronavirus-2 (SARS-CoV-2) vaccines in December 2020 [1]. Messenger ribonucleic acid (mRNA) based vaccines were the first to be authorised [1]. This was the first time these vaccines were authorised for public health use globally [2]. These were beyond the reach of many Sub-Saharan Africa (SSA) countries, especially as a number of them were not part of the COVID-19 Vaccines Global Access (COVAX) initiative. COVAX refers to an alliance of several established global health institutions that aimed to improve worldwide access to COVID-19 vaccines. The WHO called for equitable vaccine distribution, realising the considerable possibility of vaccine nationalisation and hoarding [3]. As an unprecedented historical achievement, over one billion COVID-19 vaccines had been administered by the end of March 2021, just about four months from the onset of global vaccination programmes [4]. Not surprisingly, most of these were administered in developed countries, while many countries from SSA were still to initiate vaccination. Despite substantial progress in vaccination by more powerful economies [4], SSA countries substantially remain behind, and the possibility that they will continue lagging remains high if vaccine nationalism continues [5,6].

**Figure 1:**
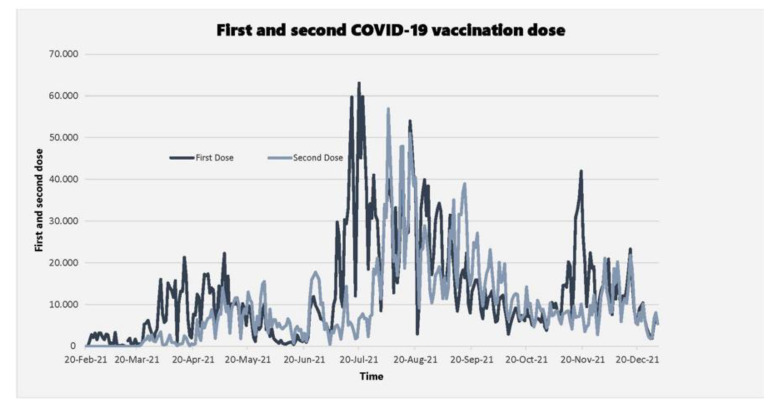
The trajectory of Zimbabwe’s national vaccine programme since its in inception in February 2021.

In realising the challenges in accessing the vaccines that had EUAs, and not being a part of the COVAX initiative, Zimbabwe sought alternative sources of vaccines. By way of donations, the country received its first batch of 200,000 doses of Sinopharm vaccines from China [7], and subsequently more donations from China, Russia and India, kick-starting an ambitious vaccine rollout programme ahead of many of its African counterparts. The country then procured more vaccines for its population from China, which has continued as the major supplier of COVID-19 vaccines to date. Though the government later approved Johnson and Johnson vaccines for use, these have not become available [8]. Remarkably, as of 5 January 2022, 7.34 million vaccine doses had been administered in Zimbabwe, with 3.18 million people (21.4% of the eligible population) fully vaccinated. The trajectory of the country’s national vaccine programme since its inception in February 2021 to December 2021 is shown in Figure 1. This is commendable progress, placing Zimbabwe ahead of its many SSA counterparts. When Zimbabwe started the vaccination programme in February 2021, none of the vaccines used had obtained EUA from the WHO. However, in March 2021, the WHO extended its EUA to include Sinopharm vaccines [9]. Subsequently, the Sinovac vaccines were also authorised by the WHO.

SARS-CoV-2 variants have continued emerging [10], and other waves of the COVID-19 pandemic are imminent. To date, variants with increased transmissibility from the ancestral wild type have emerged, namely the Beta, Gamma, Delta and more recently, in November 2021, the Omicron variant [11]. These have had properties sufficient to warrant being termed variants of concern (VOCs) by the WHO [12]. As VOCs continue to emerge, with further associated epidemic waves, the COVID-19 pandemic may continue causing marked disruption to populations’ socioeconomic well-being and health across the globe. Economic repressions, disturbances to academic learning activities and sporting activities, as well as suppression of global tourism activities are some of the negative socioeconomic consequences of the COVID-19 pandemic.

Vaccination inarguably is the best public health strategy to limit the spread of rapidly progressive infectious diseases, reduce the associated morbidity, mortality and burden to healthcare systems, and allow a return to normal life activities. Population benefits of vaccination will be realised when an adequate proportion of the population has been vaccinated for SARS-CoV-2, which could be as high as 80–95% [13]. Zimbabwe is still to reach this target, with less than 50% of the population currently fully vaccinated. To move the vaccination programme towards achieving this big but surmountable target, it is crucial to understand the strengths, weaknesses, opportunities and threats to the programme, to inform public health policy and strategy meaningfully. This review aimed to present some of the strengths, weaknesses, opportunities and threats to the Zimbabwean national COVID-19 vaccination programme since its rollout in February 2021.

## 2. Methodology

We conducted a review of the current literature on the COVID-19 vaccination program in Zimbabwe. We searched for articles published in English on the WHO website; Google Scholar and PubMed; official public health websites operated by the government of Zimbabwe; and newspaper articles written and published within Zimbabwe. We used the following keywords: COVID-19; response; COVID-19 vaccine; SARS-CoV-2 vaccine; Zimbabwe; and other subject specific terms such as hesitancy; public health communication; pharmacovigilance; surveillance; vaccine access; vaccine distribution; policy. We used the Boolean operators AND and OR to separate the keywords. For instance, the search strategy used in PubMed was (“COVID-19”[All Fields] OR “COVID-19”[MeSH Terms] OR “COVID-19 Vaccines”[All Fields] OR “COVID-19 Vaccines”[MeSH Terms] OR “COVID-19 serotherapy”[All Fields] OR “COVID-19 Nucleic Acid Testing”[All Fields] OR “COVID-19 nucleic acid testing”[MeSH Terms] OR “COVID-19 Serological Testing”[All Fields] OR “COVID-19 serological testing”[MeSH Terms] OR “COVID-19 Testing”[All Fields] OR “COVID-19 testing”[MeSH Terms] OR “SARS-CoV-2”[All Fields] OR “sars-cov-2”[MeSH Terms] OR “Severe Acute Respiratory Syndrome Coronavirus 2”[All Fields] OR “NCOV”[All Fields] OR “2019 NCOV”[All Fields] OR ((“coronavirus”[MeSH Terms] OR “coronavirus”[All Fields] OR “COV”[All Fields]) AND 2019/11/01[PubDate]: 3000/12/31[PubDate])) AND response;[All Fields] AND (“COVID-19 vaccines”[MeSH Terms] OR (“COVID-19”[All Fields] AND “vaccines”[All Fields]) OR “COVID-19 vaccines”[All Fields] OR “COVID 19 vaccine”[All Fields]) AND (“COVID-19 vaccines”[MeSH Terms] OR (“COVID-19”[All Fields] AND “vaccines”[All Fields]) OR “COVID-19 vaccines”[All Fields] OR “sars cov 2 vaccine”[All Fields]) AND hesitancy[All Fields] OR (“public health”[MeSH Terms] OR (“public”[All Fields] OR “health”[All Fields]) OR “public health”[All Fields]) AND (“communication”[MeSH Terms] OR “communication”[All Fields]) OR (“pharmacovigilance”[MeSH Terms] OR “pharmacovigilance”[All Fields]) AND (“epidemiology”[Subheading] OR “epidemiology”[All Fields] OR “surveillance”[All Fields] OR “epidemiology”[MeSH Terms] OR “surveillance”[All Fields]) AND (“vaccines”[MeSH Terms] OR “vaccines”[All Fields] OR “vaccine”[All Fields]) OR access;[All Fields] AND (“vaccines”[MeSH Terms] OR “vaccines”[All Fields] OR “vaccine”[All Fields]) OR (“supply and distribution”[Subheading] OR (“supply”[All Fields] OR “distribution”[All Fields]) OR “supply and distribution”[All Fields] OR “distribution”[All Fields]) OR (“policy”[MeSH Terms] OR “policy”[All Fields]) AND (“zimbabwe”[MeSH Terms] OR “zimbabwe”[All Fields]).

Consistent with standard literature review methodology, some steps, such appraising evidence quality (a standard step in systematic reviews) were omitted.

To allow for a well-rounded review, the information gathered was structured and is presented according to the pre-established themes, strengths, weaknesses, opportunities and threats.

## 3. Findings

**Figure 2:**
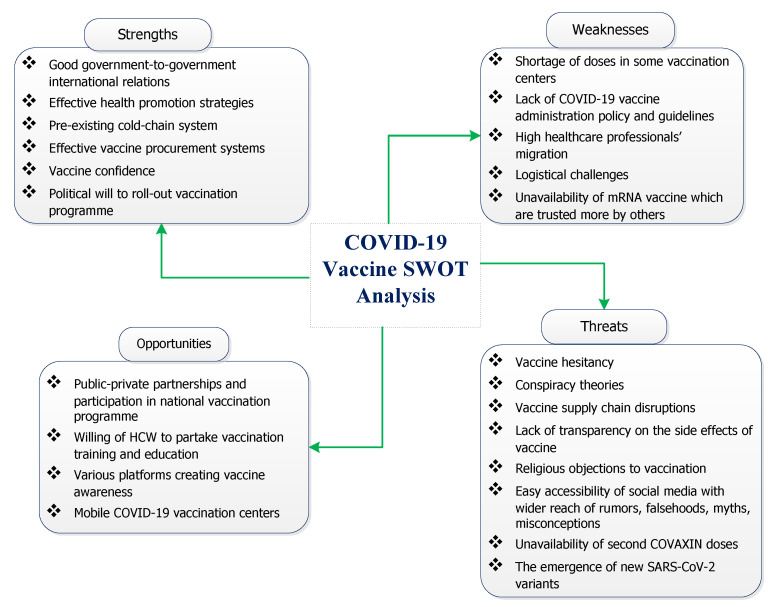
Strengths, weaknesses, opportunities and threats to the national COVID-19 vaccination programme.

The findings of this review are illustrated schematically in Figure 2.

### 3.1. Strengths

The government of Zimbabwe demonstrated its political will and commitment to the vaccination programme by looking for alternative sources of vaccines earlier on when the WHO-approved vaccines were expensive and inaccessible. Pre-existing good relations between Zimbabwe and countries such as China, India and Russia enabled the government to access vaccines early, including at least 500,000 donated doses [14]. Among the first to be vaccinated in the country was the Vice President, followed by other high-ranking officials. Healthcare workers were among the first targets of vaccination in Zimbabwe, and when some of them received the vaccines they shared their positive experiences on social and mainstream media [15]. Such activities might have helped to build up vaccine confidence and uptake by the general population. People had been largely sceptical about receiving vaccines whose safety and effectiveness profiles were largely unknown.

Zimbabwe rolled out its phased COVID-19 vaccination programme in February 2021. Phase 1 of the programme targeted healthcare workers and frontline workers such as those working at ports of entry into the country [16]. The uptake of the vaccine by healthcare workers is believed to have positively influenced the rest of the population to get vaccinated [17]. There are five vaccine choices approved for use in Zimbabwe‚ including China’s Sinovac and Sinopharm approved by WHO for emergency use‚ Russia’s Sputnik V and India’s Covaxin. This gives the population options to choose from. There are high levels of trust in the World Health Organization and the country’s Ministry of Health and Child Care (MoHCC) as sources of information. As such, the endorsement of the Sinopharm and Sinovac vaccines for emergency use by the WHO and their acceptance by the MoHCC positively impacted vaccine acceptance and uptake in Zimbabwe. With the availability of more doses, the vaccination programme entered into the subsequent phase 2 and phase 3, aiming at those with chronic conditions, other essential workers such as in the education sector, and, subsequently, the rest of the adult population above 18 years of age, provided free of charge and on a voluntary basis. Due to good relations with vaccine producing countries, the country received 943,200 COVID-19 vaccine doses from the global COVAX facility in October 2021 to supplement current national vaccine distribution [18]. The government has publicized its intentions to start vaccinating Zimbabwe’s teenagers over the age of 16 against COVID-19 in November 2021 [19]. Through their political campaigns in various parts of Zimbabwe, politicians have been trying to encourage hesitant people to get inoculated against COVID-19 amid pervasive misinformation [20]. Several organisations in Zimbabwe, including parastatals, commercial companies and public health affiliates, have embarked on an operation to encourage their members to get vaccinated amid diffidence triggered by anti-vaccination posts on social media and other platforms [17].

**Figure 3:**
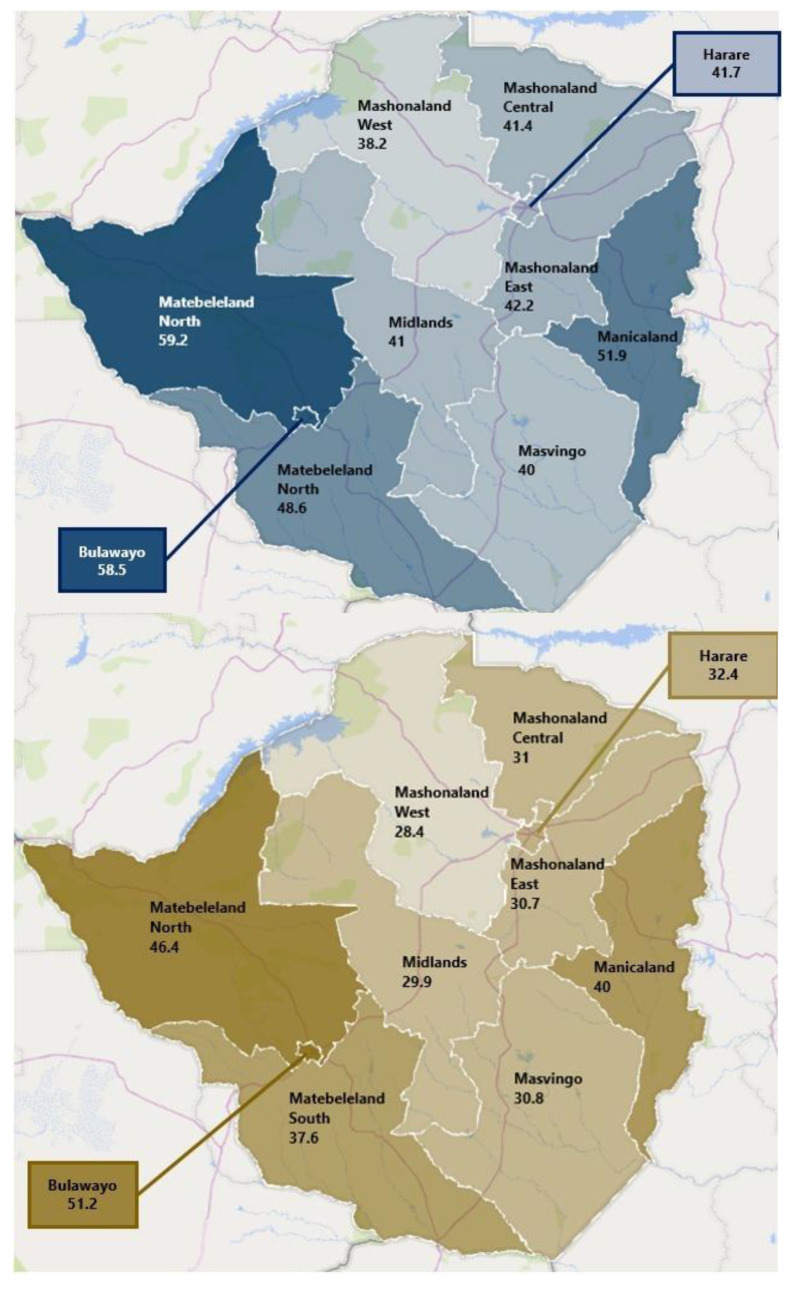
Coverage of first and second COVID-19 vaccine doses by province in Zimbabwe.

Zimbabwe has a well-run programme of expanded immunisation (ZEPI). Essential lessons to kick-start COVID-19 vaccination may have been obtained from ZEPI experiences, and the pre-existing cold-chain system provided a starting point for the national COVID-19 vaccination programme [21]. Strong collaborations with development partners such as the WHO and UNICEF are already in place, including platforms for training and monitoring and evaluation. A strong surveillance system set up by the MoHCC with support from the WHO and other partners enables close monitoring of the vaccination programme and provides vaccination statistics daily as part of the COVID-19 situation reports. The provincial coverage of COVID-19 vaccination in the country is illustrated in Figure 3 and shows that the country has made great strides in ensuring equitable distribution across the ten provinces.

### 3.2. Weaknesses

Circulating theories, mythologies, fictions and misunderstandings about the roots of COVID-19 and the intentions of vaccination continue to be big drivers of vaccine indecision and substantial obstacles to vaccine uptake in the general population [20]. Zimbabwe has vaccines from China, Russia and India but not from Western nations. Acceptance of these vaccines has been sluggish and marred with wariness of Chinese-produced vaccines, disinformation, and averseness to the vaccines by some healthcare workers and prominent government officials not disclosing their vaccination status [18]. Reported repudiations to accept the Chinese vaccines by some politicians and healthcare workers received far-reaching attention in the media and might have negatively influenced public trust in these vaccines [17]. The government has since penned a new law demanding all its employees to get vaccinated or face punitive action [22].

Vaccination centres occasionally run out of supplies, and poor urban settlements and rural areas have frequently been famished of doses [23]. Citizens have oftentimes waited for hours, only to be told the vaccination centre was closing early due to shortages of doses or administering staff [24]. Those entitled to second jabs have also complained of being turned back from centres giving preference to those pursuing a first jab. Long queues disheartened several unvaccinated residents due to the presence of few centres and the slow pace at which the healthcare workers are working to give the doses [13]. There have been pervasive social media stories of COVID-19 vaccine hesitancy amongst pregnant and breastfeeding females, proliferated by circulating mythologies, delusions and rumours concerning the safety of vaccinations in this populace [21]. Presently, there is no policy on administering COVID-19 vaccines to pregnant and breastfeeding mothers, with subsequent turning away from this population from vaccination centres. Additionally, these lack of clear admissibility procedures led to lack of uniform practices, with some centres turning away clients, whereas others were vaccinating them [25].

The population seems to have been more confident in the Western vaccines such as the Pfizer BionTech, Moderna and Astra Zeneca, none of which were available to the Zimbabwean population [26]. The supplies of the vaccines have not been consistent, with episodes of stock ruptures, and those willing to be vaccinated not able to access the vaccines. Vaccination centres were reportedly limited and faced human resource challenges. Zimbabwe has been affected by a high level of healthcare worker migration to other countries that offer better remuneration and working conditions [27]. Consequently, people have had to queue for long hours or days or move from one place to another searching for the vaccines. Sometimes, those who received the first dose have struggled to access the second dose, resulting in prolonging the period between the first and the second dose. A good proportion, close to 50% of the people who received the Covaxin vaccines from India, could not access their second dose after India ran into a harsh Delta variant driven epidemic wave and could not meet its obligations of supplying vaccines to other countries. The MoHCC did not decide on an alternative vaccine for the second dose for about six months for these clients who had failed to access their second COVAXIN dose. Logistical challenges in ensuring vaccines reach their destinations were initially widely reported [23], but this is expected with the inception of a programme, and the situation has since improved.

Lack of clear local guidelines regarding vaccination for specific groups such as the elderly, those with chronic conditions, and pregnant and breastfeeding mothers has resulted in confusion and frustration, as some were turned away from vaccination centres [25]. Similar to authorities such as the WHO and the Royal College of Obstetricians and Gynaecologists, the MoHCC must come with its guidance adapted to the local context. Lack of clear information and guidelines can propagate vaccine hesitancy. An additional group for consideration of the vaccination programme are school-going children, as currently, the vaccination programme excludes all those below 16 years of age. As calls to make the school environment safer widen, and the population is frustrated with protracted school calendar disruptions, it is critical to urgently consider this important aspect of public health [28].

### 3.3. Opportunities

The private sector in Zimbabwe is willing to participate in the national vaccination programme for very marginal profits [29]. The government must leverage this to ensure wider vaccine access to the population by ensuring those who access the private sector are vaccinated there. However, it is critical to protect the population from exploitation and profiteering by private players. Several development partners have collaborated with the government of Zimbabwe in several of its public health programmes, including HIV/AIDS, malaria, ZEPI and maternal and child health. Such partnerships can facilitate training, monitoring and evaluation, vaccine advocacy and risk communication and community engagement, and help build vaccine confidence and uptake. There is a need for a urgent conversation centred on addressing the challenge posed by vaccine uncertainties in expecting and breastfeeding women and of apparent unanimity and guiding principles to back healthcare personnel involved in administering vaccines [30]. This should be complemented by an all-encompassing education for healthcare personnel and the public to decrease misperception, upsurge confidence and increase vaccine uptake [31].

COVID-19 vaccination programs could be included in standard antenatal care procedures and baby clinics as potential solutions to attendance concerns at vaccination centres. Furthermore, antenatal clinics could give an excellent chance to provide targeted communications and counselling to expectant mothers [32]. More than half of health facilities have maternal waiting shelters, which can become possible platforms for spreading COVID-19 vaccination information through verbal communications during consultations, or through printed material in vernacular langauges that the local residents understand. Health education targeted on the safety and effectiveness of the vaccines might positively influence vaccine intentions and subsequent uptake, mainly if communication emanates from trustworthy sources [7].

Zimbabwe has several medical associations, such as the Zimbabwe College of Public Physicians and Zimbabwe Society of Obstetricians and Gynaecologists. Close collaboration between the different societies provides an opportunity to develop clear evidence-based COVID-19 vaccination consensus guidelines for special populations such as the elderly, those with chronic conditions and pregnant and breastfeeding women. Consensus guidelines can help improve vaccine uptake and the efficiency of programmes. The recent approval of the Janssen vaccines by the Medicines Control Authority of Zimbabwe is a step in the right direction. The Janssen vaccines will provide an opportunity for those members of the population who were sceptical in taking the existing vaccines, widen the basket to choose from, and provides alternatives for those who may have allergies to the current vaccines.

### 3.4. Threats

Vaccine hesitancy remains the biggest threat to the COVID-19 vaccination programmes globally [33] and is not a new phenomenon [34]. Drivers of vaccine hesitancy are contextual [31], and therefore qualitative explorations are essential to understand the drivers unique to specific populations. Zimbabwe is essentially a religious country, and religious influencers who have spoken widely against the vaccines have fuelled hesitancy. Several circulating myths, misconceptions and rumours regarding the origins of SARS-CoV-2 and the dangers of the vaccines in the population have circulated widely on diverse social media platforms, with an extensive reach [32]. Around the start of the programme in Zimbabwe, there was news of a healthcare worker who had died in Masvingo after receiving a dose of the SARS-CoV-2 vaccine, and this scared the population [35].

The emergence of SARS-CoV-2 VOCs is a global threat to vaccination efforts, including Zimbabwe [10]. VOCs have contributed significantly to subsequent epidemic waves in Zimbabwe. The second epidemic wave that occurred from December 2020 to January 2021 was mainly attributable to the Beta variant, with over 90% of the cases during the wave attributable to Beta [36]. The subsequent third epidemic wave between June and August 2021 was 98% attributable to the Delta variant [36], and though genomic sequencing results are not yet published it is postulated that the Omicron variant was the major driver of the short-lived fourth epidemic wave of COVID-19 in Zimbabwe in December 2021. In Zimbabwe and beyond, the emergence of VOCs has been a major driver of these epidemic waves as they have increased transmissibility and the potential to evade public health interventions [37,38]. The lack of adequately vaccinated populations as well as populations with compromised immunity such as people living with HIV and AIDS (PLWHA) who are prevalent in Zimbabwe and sub-Saharan Africa has been perceived as a fertile breeding ground for new VOCs and breakthrough infections among the fully vaccinated [39].

VOCs have the potential to propagate widespread community transmission, which can easily overwhelm fragile public health sectors such as in Zimbabwe. However, the greatest concern with the newer variants is their impact on vaccination programmes. VOCs may result in reduced vaccine effectiveness and shifts in herd immunity thresholds and accelerate the need for booster shots on a global scale. Evidence from other settings showed reduced effectiveness of mRNA vaccines against the delta variant, especially in terms of reducing incident infections, though the vaccines remained very effective against reducing hospitalizations and adverse outcomes from severe COVID-19 including mortality [40]. Reduced effectiveness of the chemically inactivated vaccines against the Omicron variant has been reported as well [41], and these are the mainstays of Zimbabwe’s COVID-19 vaccination programme. Messages of reduced vaccine effectiveness can reduce vaccine confidence and uptake and worsen the pre-existing vaccine hesitancy. Others have argued that natural immunity is a feasible goal with a highly transmissible variant such as Omicron and therefore do not see the need for getting vaccinated.

In the context of SARS-CoV-2, others have begun to argue that herd immunity may be an impossible goal with the continued emergence of VOCs. [37] This raises the need for continued reinvigorating of the standard infection prevention and control practices such as the wearing of facemasks, hand hygiene and physical distancing. Unfortunately, this comes at a time when pandemic fatigue and complacency among populations due to a protracted battle with the COVID-19 pandemic are on the rise, which makes effective vaccination the most critical control strategy for the pandemic. The richer countries’ demand for booster shots might lead to more vaccine hoarding and nationalisation, resulting in global shortages and supply chain disruptions. Low-income countries, including Zimbabwe, which are still to provide first doses for significant proportions of their populations, may lag behind significantly.

Zimbabweans are very mobile, primarily because of work and trade. European and other developed countries have started discussing the possibility of vaccine passports. Unfortunately, the vaccines used in Zimbabwe so far primarily have no recognition among the countries concerning the case. This may lead to those Zimbabweans intending to travel to the European Union countries and America shunning the current vaccines hoping for the approved vaccines. The approval of the Janssen vaccines is therefore welcome, but they are not yet available. The government must accelerate its efforts to procure these for the population urgently. This is more so as a heterologous approach to booster vaccination is being considered to be more superior an approach than homologous vaccination, especially against the emerging VOCs.

## 4. Recommendations

We recommend local studies to objectively identify the strengths, weaknesses, opportunities, and threats to the COVID-19 vaccination in Zimbabwe to adequately inform public health and policy. Research in the country regarding COVID-19 policy and strategies is largely insufficient. The public health stakeholders need to understand and address adequately the drivers of vaccine hesitancy in the three main categories of complacency, confidence and convenience factors as categorised by the WHO [42]. Critical to success is improving vaccine supplies, availability and accessibility, making sure that the most vulnerable groups in society are reached. The different professional medical organisations within the country must urgently come up with consensus guidelines to address especially special groups’ concerns. To these ends, the government must leverage on the existing collaborations with the different development partners that operate within and outside the country and continue strengthening the bilateral relations with countries such as China, India and Russia.

## 5. Conclusions

Though the Zimbabwe COVID-19 vaccination programme has several strengths and there are opportunities to strengthen it, vaccine hesitancy and the emergence of newer VOCs continue to pose significant threats to the current tremendous vaccination efforts in the country. There is a need to reinvigorate the vaccination programme in Zimbabwe, before further waves of COVID-19, to reduce the potential impacts of widespread community transmission on the public health sector. The public healthcare sector is highly fragile, and vaccination will serve to reduce strain by reducing hospitalisations, morbidity and mortality from COVID-19. Therefore, the government must urgently leverage on the strengths and opportunities to build vaccine confidence and uptake and address the weaknesses and threats adequately. There is an urgent need to continue procuring more vaccines to avoid stock outages, acquire other vaccines, especially the mRNA-based vaccines to widen the basket to choose for the consumers, and for strengthened messages through the various media available to build vaccine confidence and uptake. Addressing vaccine hesitancy is extremely important moving forward. Therefore, to achieve the targets, an urgent multisectoral intersection between the different relevant stakeholders in public health is required, as well as strengthening ties with the private sector. To be able to acquire other vaccine types, given the limited financial resources in the country, there is need for better cooperation with international partners, including joining pre-existing arrangements such as the COVAX initiative.

## Data Availability

All data related to this study are presented in the manuscript.
